# Evaluation of the Leishmanicidal and Cytotoxic Potential of Essential Oils Derived From Ten Colombian Plants

**Published:** 2013

**Authors:** JF Sanchez-Suarez, I Riveros, G Delgado

**Affiliations:** 1 Immunotoxicology Research Group, Dept. of Pharmacy. Faculty of Sciences, Universidad Nacional de Colombia, Bogotá, Colombia; 2 Green Andina LTDA, Bogotá, Colombia

**Keywords:** Essential oils, Antileishmanial properties, *Leishmania*, Plant, Colombia

## Abstract

**Background:**

The leishmanicidal and cytotoxic activity of ten essential oils obtained from ten plant specimens were evaluated.

**Methods:**

Essential oils were obtained by the steam distillation of plant leaves without any prior processing. Cytotoxicity was tested on J774 macrophages and leishmanicidal activity was assessed against four species of *Leishmania* associated with cutaneous leishmaniasis.

**Results:**

Seven essential oils exhibited activity against *Leishmania* parasites, five of which were toxic against J774 macrophages. Selectivity indices of >6 and 13 were calculated for the essential oils of *Ocimum basilicum* and *Origanum vulgare*, respectively.

**Conclusion:**

The essential oil of *Ocimum basilicum* was active against promastigotes of *Leishmania* and innocuous to J774 macrophages at concentrations up to 1600 µg/mL and should be further investigated for leishmanicidal activity in others *in vitro* and *in vivo* experimental models.

## Introduction

Leishmaniasis is a disease caused by protozoan parasites of the genus *Leishmania*, which are endemic in 88 countries, 72 of which are developing countries (1). Currently, leishmaniasis affects more than 12 million people worldwide, and 350 million people are estimated to be at risk of contracting this disease. Each year, approximately 2 million new cases of infections are reported worldwide (2). During the course of the *Leishmania* spp. infection, the following three well-defined clinical pictures can manifest: cutaneous leishmaniasis (CL), mucosal leishmaniasis (ML) and visceral leishmaniasis (VL). The latter usually has a higher reported mortality. The progression of one of these clinical forms depends on several variables, such as the species of *Leishmania*, the vector involved in the infection, the strain of the parasite and the immune and nutritional status of the individual (3).

Leishmaniasis is currently treated by chemotherapy with pentavalent antiminioles salts (first-line drugs), which recently has reported an increase in cases of therapeutic failure (4–6), and although there are other treatment options such as miltefosine, which has the advantage of oral administration and low toxicity, there is no evidence that miltefosine outrank meglumine antimony (7–8). As a result, the search for new active antileishmanials is imperative and has been promoted by WHO, which endorses the use of traditional medicine (natural products) when appropriate health services are inaccessible (9). Within the group of natural products, essential oils have been of interest due to their broad spectrum of reported biological activities (10–12), including leishmanicidal activity (13–16). In addition, their hydrophobic nature makes these oils more permeable to cells (17), which is a very important feature for developing agents against intracellular pathogens.

The present study evaluated the leishmanicidal and cytotoxic effects of ten essential oils obtained from the following species of Colombian plants: *Ocimum basilicum* L. (basil, Eo-1), *Zingiber officinale* Roscoe (ginger, Eo-2), *Citrus limon* (L.) Burm. f. (lemon, Eo-3), *Cymbopogon citratus* (DC.) Stapf (lemongrass, Eo-4), *Mentha x piperita* L. */ M. pulegium* L. (50/50). (mint, Eo-5), *Citrus sinensis* (L.) Osbeck (orange, Eo-6), *Origanum vulgare* L. (oregano, Eo-7), *Rosmarinus officinalis* L. (rosemary, Eo-8), *Thymus vulgaris* L. (thyme, Eo-9) and *Coriandrum sativum* L. (coriander, Eo-10). From the selected plants, four have antitrypanosomal activity reports [*Ocimum basilicum* (18), *Zingiber officinale* (19) *Citrus sinensis* (20) and *Thymus vulgaris* (21)], five reported antimicrobial effect [*Citrus limón* (22) *, Mentha x piperita* L. (23), *M. pulegium* L. (23) *, Rosmarinus officinalis* (24) *, Coriandrum sativum* (25)], and finally for two had reports of both antitrypanosomal and antimicrobial activities [*Cymbopogon citratus* (20, 26–27) *, Origanum vulgare* (21, 28)]. The antileishmanial effect was evaluated against promastigotes of *Leishmania major, L. panamensis, L. braziliensis, L. guyanensis* and murine macrophages (J774 cell line) and taking into account that *Leishmania* is an intracellular parasite, the cytotoxic potential of any compound with leishmanicidal properties must be evaluated in the cell targeted for parasite infection keeping in mind the above, the cytotoxic effect was evaluated on J774 macrophage cell line.

## Materials and Methods

### Cell cultures

The J774 macrophage cell line was cultured in sterile 25 cm^2^ plates (Techno Plastic Products AG, Switzerland) in RPMI-1640 medium (Gibco BRL-Life Technologies Inc., Grand Island, NY, USA) supplemented with 5% fetal bovine serum (Microgen, LTDA, Bogota, Colombia) and incubated in a CO_2_ (5%) incubator at 37°C.

The following four strains of promastigotes were maintained in sterile 25 cm^2^ culture plates (Techno Plastic Products AG, Switzerland) containing RPMI-1640 medium (Gibco BRL-Life Technologies Inc., Grand Island, NY, USA) supplemented with 5% of fetal bovine serum (Microgen, LTDA, Bogota, Colombia) and 2 mM L-glutamine (Gibco BRL-Life Technologies Inc., Grand Island, NY) under ambient humidity and gasification at 26 °C: *L. major* (Friedlin V1 strain), which was kindly donated by Dr. Jimena Cortés from the Autonomous University of Madrid (Universidad Autónoma de Madrid), Spain, and *L. braziliensis* (MHOM/CO/2011/UA3320), *L. guyanensis* (MHOM/CO/84/CL-007) and *L. panamensis* (MHOM/CO/98/UA1702), which were kindly donated by Dr. Sara Robledo from Antioch University.

### Extraction of essential oils/Plant materials

Plants were collected from Green Andina crops LTDA in the municipality of Tena, Cundinarmarca department (Colombia) under industrial conditions according to the company's protocol.

### Obtaining essential oils

The essential oils were obtained by the steam distillation of plant leaves without any prior processing, with the exception of the essential oil of coriander (Eo-10), which was obtained from *Coriandrum sativum* seeds.

### Cytotoxicity assays

To evaluate the susceptibility of J774 macrophages to essential oils, 10^4^ cells/well were seeded in a flat bottom 96-well plate (Techno Plastic Products AG, Switzerland) and incubated for 18 to 24 hours for optimal adhesion. The J774 macrophages were then exposed to four different concentrations of the essential oils (between 1600 µg/mL and 25 µg/mL of each essential oil) and incubated in 5% CO_2_ for 72 hours at 37 °C. Then, resazurin was added to the cells for a final concentration of 44 µM (29). After four hours, the reduction of resazurin to resorufin was monitored using a Tecan GENios Microplate Reader (Tecan, Austria) with the software Magellan version 4.0 (Tecan, Austria) at excitation and emission wavelengths of 535 and 590 nm, respectively. The assays were conducted at two different times and each in duplicate.

### Leishmanicidal activity assays on promastigotes

The *Leishmania* strains (2 x 10^5^ parasites) were each seeded into a 96-well flat bottom plate containing four different concentrations of essential oils (between 640 µg/mL and 10 µg/mL of each essential oil). Samples were incubated for 72 hours followed by the addition of 50 µL of RPMI containing 220 µM resazurin to each well for a final volume of 250 µL and resazurin concentration of 44 µM. After 36 hours, the reduction of resazurin to resorufin was monitored by measuring spectrofluorometric emission using the Tecan GENios Microplate Reader equipped the software Magellan version 4.0. The same form that cytotoxic test, the leishmanicidal assays were conducted at two different times and each in duplicate.

In both cytotoxicity and leishmanicidal activity assays, negative control cells were macrophages and parasites only exposed to the medium. Positive control cells were exposed to different concentrations of the leishmanicidal reference drug pentamidine isethionate (Pentacarinat ^®^, Sanofi, Aventis).

### Statistical analysis

Data were normalized to cells without treatment to determine the percentage of survival. Inhibitory concentrations (lethal concentration 50 [LC_50_] on macrophages and effective concentration 50 [EC_50_] on parasites) were calculated by the software GraphPad Prism v5.0 (GraphPad Software, USA) using a nonlinear regression model of variable slope. *P* values less than 0.05 were considered to be significant.

## Results

Table 1 shows the results of the cytotoxicity assays and leishmanicidal effects of the 10 essential oils on *Leishmania* spp. Promastigotes. In regard to the cytotoxic potential of essential oils tested, it was only possible to calculate LC_50_ for four. Two of these (Eo-9 and Eo-10) exhibited LC_50_ above 400 µg / mL, concentrations which are relatively high and can be considered as low cytotoxicity. While the antileishmanial activity, only three (Eo-5, Eo-6 y Eo-8) essentials oils failed to inhibit the viability of at least one species of *Leishmania* parasites. Of the remaining seven, Eo-4 showed leishmanicidal activity between 149 and 180 µg/mL. For the rest of the tested substances, the activity varied depending on the species of parasite which was exposed. Eo-7 was the most potent essential oil as it showed the lowest EC_50_ calculated in this study.

**Table 1:** Leishmanicidal and cytotoxic activities of the essential oils evaluated

CODE	J774	*L. panamensis*		*L. braziliensis*			*L. major*		*L. guyanensis*
	LC_50_ a	EC_50_ b	SI^c^	EC_50_	SI	EC_50_	SI	EC_50_	SI
Eo-1	>1,600 ± 0.0	251.59 ± 64.18	>6,4^d^	>640 ± 0.0	NC	>640 ± 0.0	NC	315.55 ± 90.86	>5 d
Eo-2	156.1 ± 40.87	154.83 ± 23.86	1,0	124.94 ± 52.98	1,2	303.0 ± 107.48	0,5	256.95 ± 75.17	0,6
Eo-3	>1,600 ± 0.0	>640 ± 0.0	NC	>640 ± 0.0	NC	>640 ± 0.0	NC	231.4 ± 42.43	>6,9 d
Eo-4	214.7 ± 47.98	180.83 ± 82.24	1,2	160.06 ± 43.49	1,3	194,05 ± 29.20	1,1	149.1 ± 6.22	1,4
Eo-5	>1,600 ± 0.0	>640 ± 0.0	NC	>640 ± 0.0	NC	>640 ± 0.0	NC	>640 ± 0.0	NC
Eo-6	>1,600 ± 0.0	>640 ± 0.0	NC	>640 ± 0.0	NC	>640 ± 0.0	NC	>640 ± 0.0	NC
Eo-7	544.6 ± 26.30	42.23 ± 2.04	12,9	204.36 ± 21.56	2,7	171.8 ± 20.64	3,2	>640 ± 0.0	<1^e^
Eo-8	>1,600 ± 0.0	>640 ± 0.0	NC	>640 ± 0.0	NC	>640 ± 0.0	NC	>640 ± 0.0	NC
Eo-9	434.9 ± 133.36	402.23 ± 82.90	1,1	>640 ± 0.0	<0,7^e^	>640 ± 0.0	<0,7^e^	>640 ± 0.0	<0,7^e^
Eo-10	1,267.9 ± 133.36	427.95 ± 118.44	3,0	>640 ± 0.0	<2,0^e^	>640 ± 0.0	<2,0^e^	>640 ± 0.0	<2,0^e^
Pentamidine	4.64 ± 2.62	0.049 ± 0.004	94,7	0.65 ± 0.28	7,1	0.24 ± 0.004	19,3	0.06 ± 0.002	77,3

*Ocimum basilicum* L. (basil, Eo-1), *Zingiber officinale* Roscoe (ginger, Eo-2), *Citrus limon* (L.) Burm. f. (lemon, Eo-3), *Cymbopogon citratus* (DC.) Stapf (lemongrass, Eo-4), *Mentha x piperita* L. / *M. pulegium* L. (50/50). (mint, Eo-5), *Citrus sinensis* (L.) Osbeck (orange, Eo-6), *Origanum vulgare* L. (oregano, Eo-7), *Rosmarinus officinalis* L. (rosemary, Eo-8), *Thymus vulgaris* L. (thyme, Eo-9) and *Coriandrum sativum* L. (coriander, Eo-10).

a Lethal concentration 50 (µg/mL) ± standard deviation.

b Effective concentration 50 (µg/mL) ± standard deviation.

c Selectivity index (LC_50_/EC_50_)

d Since the LC_50_ was not exactly determined, the IS is reported as “greater than” the calculated

e Since the EC_50_ was not exactly determined, the IS is reported as “less than” the calculated

NC, Not Calculated.For those assays where we could not determine the LC_50_ and EC_50_.

## Discussion

Of all the oils that were active against promastigotes (70% of oils evaluated), the following three oils were active against one strain: Eo-3 (against *L. guyanensis*), Eo-9 (against *L. panamensis*) and Eo-10 (against *L. panamensis*). Notably, Eo-3 was innocuous in cytotoxicity assays with a selectivity index (SI) greater than 7. Eo-3, which is the essential oil derived from *Citrus limon*, is currently used as an alternative therapy in Brazilian communities for the treatment of leishmaniasis (30). In addition, the antiparasitic activity of this oil has been demonstrated against flagellates *in vitro* (31).

Eo-1 was determined to be active against *L. panamensis* and *L. guyanensis* with an SI greater than 5. Similar to Eo-3, Eo-1 exhibited anti-leishmanial effects and relatively high concentrations of this oil were required to show deleterious effects on the cellular viability of macrophages. The inhibitory effects of the *Ocimum basilicum* essential oil (Eo-1) has previously been evaluated on *L. donovani*, and EC50 values between 37.3 and 49.6 µg/mL were reported for plant varieties from the U.S (15), which are well below that measured for Eo-1 in the present study. However, *L. donovani* is a different species and the composition of plant extracts (as in the case of essential oils) can vary depending on various environmental and geographical factors (32).

Eo-7 was one of the most active oils, exhibiting leishmanicidal effects against the following three strains tested: *L. panamensis, L. braziliensis* and *L. major*. The inhibitory activity of the oregano essential oil (Eo-7) against bacteria (33) and *Trypanosoma cruzi* (*Leishmania* parasites belonging to the Trypanosomatidae family) (21) has been reported. Only Eo-2 and Eo-4 were capable of inhibiting the cell viability of macrophages and all four strains of parasites above 50%. Mild antileishmanial activity of aqueous and ethanol extracts of *Zingiber officinale* on *L. chagasi* and *L. Mexicana* has been reported (34). In addition, antileishmanial and significant antitripanosomial activity was detected in a curcuminoid isolated from *Z. officinale* (35). *Cymbopogon citratus* has also been reported to exhibit leishmanicidal activity against *L. amazonensis* (36) and *L. chagasi* (37) *in vitro*. However, these effects were observed within the same range of concentrations for cytotoxic activity (SI = 1), limiting the leishmanicidal potential of the essential oils from *Cymbopogon citratus*.

The reference drug exhibited a differential effect on *Leishmania* species. For example, *L. braziliensis* was the least susceptible to pentamidine isethionate, whereas *L. panamensis* and *L. guyanensis* were the most susceptible. This observation is not surprising considering that some studies divide the subgenus *Viannia* into complexes of species in which *L. panamensis* and *L. guyanensis* are grouped together and *L. braziliensis* is an independent complex (38).

Eo-2 and Eo-4 were the most active essential oils against all four species of parasites tested and should be further evaluated to identify metabolites with a broad spectrum of activity, even though they may not be equally active against host cells. The activity exhibited by Eo-1 and Eo-7 were the most promising, as evidenced by their SI values. However, Eo-1 appears to be the most active essential oil because it lacked significant cytotoxic effects and exhibited similar leishmanicidal activity against two closely related species (38) that are of clinical importance in Colombia (39–40). Because cutaneous leishmaniasis is the predominant clinical form of this disease in Colombia, a topical formulation is the alternative treatment of choice. Due to their organoleptic and oiliness properties of essential oils, their direct application to ulcers and skin lesions would be favored. However, even though the oils described here are promising antileishmanials, their leishmanicidal effects on intracellular amastigotes need to be further investigated.

## Conclusion

The present work demonstrates leishmanicidal activity of seven essential oils, of which the more potent were Eo-2, Eo-4 and Eo-7, because they showed the broader spectrum of antileishmanial action. However, Eo-7 was the only one that exhibited a selective effect, which was larger against *L. panamensis*.
